# Bacterial Genetic Architecture of Ecological Interactions in Co-culture by GWAS-Taking *Escherichia coli* and *Staphylococcus aureus* as an Example

**DOI:** 10.3389/fmicb.2017.02332

**Published:** 2017-11-27

**Authors:** Xiaoqing He, Yi Jin, Meixia Ye, Nan Chen, Jing Zhu, Jingqi Wang, Libo Jiang, Rongling Wu

**Affiliations:** ^1^Center for Computational Biology, College of Biological Sciences and Technology, Beijing Forestry University, Beijing, China; ^2^College of Biological Sciences and Technology, Beijing Forestry University, Beijing, China; ^3^Center for Statistical Genetics, Pennsylvania State University, Hershey, PA, United States

**Keywords:** bacterial interactions, bacterial phenotypes, whole-genome sequencing, genome-wide association studies, significant SNPs

## Abstract

How a species responds to such a biotic environment in the community, ultimately leading to its evolution, has been a topic of intense interest to ecological evolutionary biologists. Until recently, limited knowledge was available regarding the genotypic changes that underlie phenotypic changes. Our study implemented GWAS (Genome-Wide Association Studies) to illustrate the genetic architecture of ecological interactions that take place in microbial populations. By choosing 45 such interspecific pairs of *Escherichia coli* and *Staphylococcus aureus* strains that were all genotyped throughout the entire genome, we employed Q-ROADTRIPS to analyze the association between single SNPs and microbial abundance measured at each time point for bacterial populations reared in monoculture and co-culture, respectively. We identified a large number of SNPs and indels across the genomes (35.69 G clean data of *E. coli* and 50.41 G of *S. aureus*). We reported 66 and 111 SNPs that were associated with interaction in *E. coli* and *S. aureus*, respectively. 23 out of 66 polymorphic changes resulted in amino acid alterations.12 significant genes, such as *murE, treA, argS*, and *relA*, which were also identified in previous evolutionary studies. In *S. aureus*, 111 SNPs detected in coding sequences could be divided into 35 non-synonymous and 76 synonymous SNPs. Our study illustrated the potential of genome-wide association methods for studying rapidly evolving traits in bacteria. Genetic association study methods will facilitate the identification of genetic elements likely to cause phenotypes of interest and provide targets for further laboratory investigation.

## Introduction

Understanding species’ adaptation to new environments is essential for elucidating biosystem dynamics and for predicting responses to changes imposed by humans on the environment (Lawrence et al., 2012). When faced with environmental changes, species interactions not only give rise to ecological changes in species’ abundances and distribution but also affect how component species evolve. This, in turn, affects ecosystem functioning (Brown et al., 2001). There are multiple examples where co-evolution accelerates the evolution of a species (Brockhurst et al., 2003), and species coevolution differs from evolution when species are grown in isolation (Schluter et al., 1985). In the macroecological world, co-evolution between competitors, mutualists, etc., has been repeatedly observed (Hibbing et al., 2010). Although multiple ecological mechanisms have been proposed to explain why species interactions facilitate adaptation to environmental changes (Turcotte et al., 2012), the genetic underpinnings remain unknown.

Microorganisms have short generation times, large population sizes, and the ability to preserve ancestor strains (Elena and Lenski, 2003; Conrad et al., 2009), making them ideal systems for studying adaptive evolution. By culturing five species of bacteria separately or in mixtures in a new environment for many generations, Lawrence et al. (2012) observed that each species adapted more rapidly in mixed cultures than in monocultures. However, few studies have discussed co-evolution in mixed-species environments of bacteria (Herring et al., 2006). Intermicrobial competition occurs in many natural environments, and such interactions are important for the regulation of multiple biogeochemical processes (Bell et al., 2013). An integrated description of environmental interaction networks is lacking, although it is necessary to understand the medical and ecological consequences of bacterial communities (Freilich et al., 2010).

Until recently, limited knowledge was available regarding the genotypic changes that underlie phenotypic changes (Plata et al., 2015). To gain additional insight, studies of genome evolution require whole-genome sequencing (WGS) combined with microbiological experimentation (Barrick and Lenski, 2013). Adaptive laboratory evolution is a growing field that has been advanced by WGS (LaCroix et al., 2015). Currently, WGS is used to identify genomic variants that underlie phenotypic variations, adaptation, etc., in natural populations (Gouin et al., 2015). Thus, WGS allows for analysis of the structure and content of microbial genomes more comprehensively than has previously been possible (Holt et al., 2008; Ford et al., 2011; Berscheid et al., 2012; Forde and O’Toole, 2013). In contrast to traditional sequencing methods, WGS offers information about the genetic basis of phenotypic traits by identifying single nucleotide differences (Price et al., 2013). WGS of microbes enables the discovery of essential parameters of adaptive evolution in bacteria, including the number of mutations, the functions of the mutated genes, etc. However, few investigations of adaptive evolution in bacteria have included genome resequencing (Conrad et al., 2009).

Next-generation sequencing (NGS) facilitates the identification of single nucleotide polymorphisms (SNPs) (Conrad et al., 2009; Kanesaki et al., 2012), with the SNPs derived being used to dissect the genomic characteristics of microbes. For example, Ojeda et al. (2014) developed a road map to discover SNPs for population genomics studies in the fungal symbionts of the mountain pine beetle using WGS technology. The first complete genome sequence for *Escherichia coli*, K-12 MG1655, was publicly released in 1997, of which the 4,639,221-base pair sequence was presented (Blattner et al., 1997). Since then, real-time WGS has been applied for genotyping and outbreak detection of verotoxigenic *E. coli* (Joensen et al., 2014). Additionally, Knobloch et al. (2014) used WGS to assess the long-term risk of Shiga toxin-producing *E. coli* carriage in patients. *Staphylococcus aureus* is a leading cause of hospital- and community-acquired infection worldwide. The first complete *S. aureus* genome was published in 2001 (Kuroda et al., 2001), after which Sabirova et al. (2014) used WGS to rapidly assess the genomic stability of key reference strains.

Genome-wide association study (GWAS) involves testing large numbers of genetic variants, usually SNPs or insertions and deletions (indels) within a population of individual organisms, for associations with a given phenotype (Read and Massey, 2014). GWAS facilitates the identification of genetic elements that are likely to cause phenotypes of interest and to provide investigational targets (Aulchenko et al., 2007). The first successful GWAS in humans was published in 2005 and examined 96 patients with age-related macular degeneration, a condition that leads to vision loss in older adults, and 50 age-matched controls (Klein et al., 2005; Read and Massey, 2014). GWAS can identify genetic factors underlying important phenotypes, but have rarely been applied to bacteria (Sheppard et al., 2013; Buchanan et al., 2017). The application of GWAS to microorganisms is a potentially powerful approach to rapidly identifying genetic factors that mediate heritable phenotypic variation (Muller et al., 2011; Connelly and Akey, 2012; Dalman et al., 2013). GWAS by collecting a random sample of genotypes from a natural or experimental population provides a framework for studying the genetic architecture of complex traits. However, the possible confounding effect of population stratification on GWAS analysis should be removed to correctly infer the genetic basis of bacterial phenotypes (Alam et al., 2014; Read and Massey, 2014; Chen and Shapiro, 2015). Thornton and Mcpeek (2010) developed an approach, named ROADTRIPS, for performing GWAS in a case-control setting, which can account for any unknown population structure or relatedness within samples. More recently, this approach has been extended to analyze quantitative traits in a population-based GWAS setting, known as Q-ROADTRIPS^1^.

*Escherichia coli* and *S. aureus* are powerful model organisms for genome-wide studies because of their relatively small genome sizes. In this study, we cultured different pairs of *E. coli* and *S. aureus* strains in the same media, and the abundance of each strain was observed at different time points. Following this, GWAS was performed to identify genes that regulated specific ecological processes in the artificial co-culture. From the data, we characterized specific genes that mediated evolution during co-culture.

## Materials and Methods

### Flow Cytometry

For total cell counts, an aliquot of 10 μL of SYBR^®^Green I (Invitrogen, United States), 100-times diluted in DMSO (Sigma, United States), and mixed with PI (0.6 mM final concentration) was added to 1 mL of a suspension and incubated for 10 min at 35°C in the dark before analysis. For better permeabilization of the outer membrane EDTA (pH 8) was added (5 mM final concentration) to the sample together with the stain.

All samples were measured on a FACSCalibur instrument (BD FACSCalibur, United States) equipped with an argon laser emitting at a fixed wavelength of 488 nm and equipped with CellQuest hardware. The trigger was set on the green fluorescence (520 nm) channel and signals for total cell counting were collected on the combined 520 nm/630 nm (red fluorescence from SYBR Green) dot plot (Hammes et al., 2008).

### Bacterial Strains and Pre-cultivation

We collected 45 strains of *E. coli* and 45 strains of *S. aureus* from National Infrastructure of Microbial Resources, China (**Supplementary Table S1**). Although all the bacterial strains were not pathogenic, we still deal with them very carefully in Biosafety level two laboratories. The cryo-cultures were streaked onto a Tryptic soy agar plate and incubated for 24 h at 37°C. Cells from a single colony were transferred with a loop into Nutrient Broth (OXOID, Basingstoke, England) and were incubated overnight at 30°C. Subsequently, cells from this overnight culture were transferred into 10-times diluted Nutrient Broth medium (starting concentration 5×10^3^ cells/mL by FCM) and incubated for 4 days at 30°C before used as inoculums.

### Pairwise Evolution Experiments

Cultures were established in 50 ml flasks containing 20 ml of 10-times diluted Nutrient Broth and inoculated initially from established cultures of bacteria after 4-days cultivation. In ‘monoculture’ treatments, cultures were started with each species in the 20 ml medium, respectively (starting concentration is 5 × 10^3^ cells/mL by FCM). During ‘co-culture’ treatments, inoculates of each of the species was added to the same flask to create a two-species community of bacteria. The starting concentration of each species is 5 × 10^3^ cells/mL by FCM).

Sixteen replicates of each species in monoculture and of each two-species pair were set up following the protocol in **Supplementary Figure S1**. We performed three replicates for both monoculture and co-culture. Experiments started with two strains in monoculture or in co-culture (two strains mixed together). The flasks were incubated at 30°C and shaken at 130 rpm. To stimulate active growth and promote adaptation to the low concentration conditions, each culture was diluted 10-fold in fresh medium twice weekly for 8 weeks. Every 3 or 4 days, 1 mL from each microcosm was transferred to 19 mL fresh media. Flasks were shaken to prevent the formation of biofilms and maintain spatial homogeneity. Cultures at each time point were measured by qPCR method. The final cultures were incubated at 30°C to select single colonies by plating on Nutrient Broth agar. Final isolates were stored at -80°C for use in subsequent assays.

### Maximum Growth Rates of Monoculture Isolates and Co-culture Isolates

Species were recoverable from stored frozen final isolates and were used for the measurement of maximum growth rate, which were performed in 20 mL of Nutrient Broth in 50 mL flasks inoculated with 200 μL of bacteria. The flasks were incubated at 30°C with shaking at 130 rpm. OD_600_ values were measured per hour using the Microplate reader (Infinite M200 PRO, TECEN, Switzerland). Readings were subtracted from negative controls of sterile medium placed on each column of the plate. The statistical analysis of maximum growth rates was performed using SPSS 12.0 (SPSS Taiwan Corp., Taiwan). Data followed by the same letters were not significantly different based on Duncan’s multiple range tests at *p* ≤ 0.05. Each process was repeated three times.

### qPCR

Quantitative real-time PCR was performed in an Mx3005P realtime quantitative PCR system (Stratagene, La Jolla, CA, United States) in a total volume of 25 μL, consisting of the SuperReal PreMix Plus (SYBR Green) (TIANGEN, Beijing, China), 300 nM forward primers and 300 nM reverse primers. For specific detection of *E. coli* species, 217 bp of the regulatory region of *uidA* gene, designated *uidR*, which is located upstream of the *uidA* structural gene, were amplified by forward primer GTGGCAGTGAAGGGCGAACAGT and reverse primer GTGAGCGTCGCAGAACATTACA (Bej et al., 1991). For specific detection of *S. aureus* species, 226 bp of *nuc* gene encoding thermostable nuclease, were amplified by forward primer AAAGGGCAATACGCAAAGAGGT and reverse primer CTTTAGCCAAGCCTTGACGAAC (Alarcón et al., 2006). Control samples, without template DNA, were also included in the runs. The thermal cycling conditions were as follows: an initial denaturation at 95°C for 10 min followed by 40 cycles of 30 s at 95°C, 1 min at 55°C, and 1 min at 72°C. Each run ended with a melting curve analysis. Fluorescence data were collected at the end of each cycle and determination of the cycle threshold line was carried out automatically by the instrument. The qPCR results of each strain/pair were the means over three independent experiments. The DNA copy number of each species was calculated using a uidA/nuc–containing plasmid of known concentration as a standard.

### Whole-Genome Sequencing

Whole-genome sequencing was performed on the Illumina HiSeq2000/2500/4000 platform at Novogene (Novogene, Beijing, China) using *E. coli* str. K-12 substr. MG1655 and *S. aureus* subsp. *aureus* NCTC 8325 as the reference strain, respectively.

#### DNA Sequencing

Genomic DNA was isolated using the DNeasy Blood and Tissue kit (Qiagen, Hilden, Germany) according to the manufacturer’s instructions. All the genomic sequencing libraries were prepared according to the manufacturer’s instructions. Forty-five *E. coli* and 45 *S. aureus* genomes were paired-end re-sequenced using the Illumina HiSeq 2000/2500/4000 instrument (Illumina inc., San Diego, CA, United States) with around 20× coverage for each genome. The initial strains in this experiment were genomes sequenced. Genomic DNA from different isolates were all genomes sequenced independently. Reads were 100/125/150 bp long, sequenced in pairs with a mean insert size of 500 bp. All reads were filtered out the adaptor sequences, low-quality reads and duplicate reads to get clean reads before mapping.

#### Reads Mapping and Quality Control

All reads were filtered out the adaptor sequences, low-quality reads and duplicate reads before mapping. Paired reads were discarded if the number of *N*’s in either of the paired reads exceeded 10%. Also, the number of low quality (*Q* ≤ 38) bases in a single read was restricted to less than 40%. Duplicated reads were removed. Sequencing coverage and depth for each sample were calculated.

#### SNPs Detection from Sequence Data

Illumina reads were mapped directly to the *E. coli* and *S. aureus* reference sequences using BWA mapper (Version 0.7.8; aln -e 10 -l 32 -i 15 -q 10). In alignment results, PCR duplicates were removed by SAMtools software package (Version 0.1.18; -m 2 -F 0.002 -d 1000). We also used SAMtools to detect SNPs. Every time a mapped read shows a mismatch from the reference genome, SAMtools can be used to figure out whether the mismatch is due to a real SNP. It incorporates different types of information, such as the number of different reads that share a mis-match from the reference, the sequence quality data, and the expected sequencing error rates, and it essentially figures out whether it’s more likely that the observed mismatch is due simply to a sequencing error, or because of a true SNP. SNPs with high quality score (*Q*-value ≥ 20), and enough supporting bases (≥4) (with the variant) were kept as final SNPs result.

Indel detection from sequence: After reads mapping using BWA mapper (Version 0.7.8; aln -e 10 -l 32 -i 15 -q 10), a similar short indel detection (≤50 bp) was performed by SAMtools software package (Version 0.1.18; -m 2 -F 0.002 -d 1000). Indels with high quality score (*Q*-value ≥ 20), and enough supporting bases (≥4) (with the variant) were kept as final indels result.

### Genome-Wide Association Study (GWAS)

In this study, we employed Q-ROADTRIPS to analyze the association between single SNPs and microbial abundance measured at each time point for bacterial populations reared in monoculture and co-culture, respectively. By analyzing the association of each SNP with the abundance, Q-ROADTRIPS calculated the *P*-values of each association, from which to make Manhattan plots of GWAS for two different species, at different time points and under the two treatments, monoculture and co-culture, respectively. The genome-wide significance level was then determined through adjusting for multiple comparison using the Bonferroni correction approach I.

We have also identified a set of insert/deletion (indels), which represent a different type of genetic variants throughout the microbial genomes. The associations of each indel variant with microbial abundance for two different species, at different time points and under different treatments were also analyzed, tested and corrected using a regression model.

## Results

### Maximum Growth Rates of Initial Strains and Isolates in Monoculture and Polyculture

**FIGURE 1:**
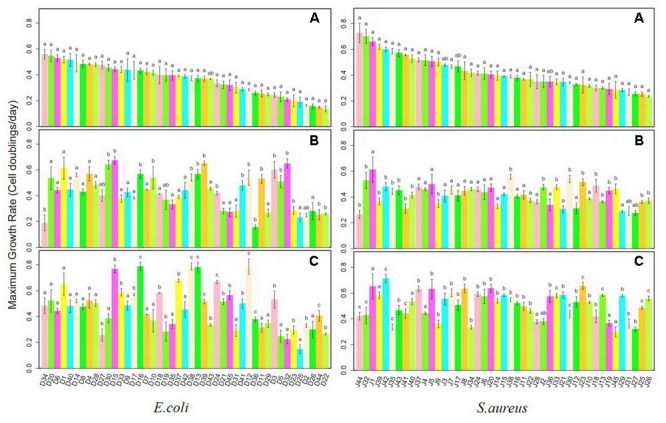
Maximum growth rates for *Escherichia coli* and *Staphylococcus aureus* strains after monoculture and co-culture. **(A)** Initial Strains; **(B)** monoculture; **(C)** co-culture.

Growth rates for each *E. coli* and *S. aureus* strain were determined in monoculture and interspecific co-culture, compared with those of initial strains. Isolates from the two species were ordered according to values of the maximum growth rates of initial strains from largest to smallest. In **Figure 1**, there were two *E. coli* isolates (D34, D36) growing significantly slower in monoculture than initial isolates of the same species and 17 isolates (D30, D15, D16, D10, D38, D13, D39, D24, D41, D12, D11, D3, D5, D32, D23, D44, D22) growing significantly faster. Nine *S. aureus* isolates (J44, J32, J39, J42, J35, J43, J41, J40, J9) in monoculture had significantly slower growth rates than initial isolates (J38, J2, J33, J30, J23, J18, J13, J19, J45, J25, J26) while 11 isolates had significantly faster growth rates. Twenty *E. coli* isolates (D15, D33, D17, D16, D18, D37, D38, D13, D39, D24, D21, D45, D42, D12, D36, D3, D23, D2, D44, and D22) grown in co-culture had significantly faster growth rates than initial strains. The growth rates of 14 *E. coli* isolates (D33, D17, D16, D18, D37, D38, D13, D39, D24, D21, D45, D12, D36, and D44) in co-culture differed significantly from both those of initial strains and monoculture isolates. Among them, all but one (D39) grew faster both than initial strains and those isolated from monoculture, which may indicate that interactions in co-culture increase growth rates of *E. coli*. The growth rates of 27 *S. aureus* isolates (J44, J32, J42, J35, J41, J37, J5, J7, J8, J34, J24, J6, J20, J14, J15, J16, J11, J22, J33, J21, J30, J12, J23, J13, J29, J25, and J26) in co-culture differed significantly from those of initial strains and monoculture isolates. Five *S. aureus* strains (J44, J32, J35, J41, and J34) grew more slowly in co-culture than the initial stains. But 21 *S. aureus* co-culture isolates grew much faster than initial and monocultured isolates, which may be also the result of growth adaptations during co-culture.

### SNPs and Indels Detection

The resequencing statistics for *E. coli* and *S. aureus* genomes are summarized in **Supplementary Tables S2, S3**. To ensure high-quality SNPs and indels data from all samples, each sample was sequenced with around 20-fold coverage (**Supplementary Table S4**). After mapping the reads to the reference genome, erroneous reads caused by polymerase chain reaction duplications (<1%) were removed. The raw sequence data of *E. coli* generated in this study were deposited in the NCBI short reads archive under Accession No. SRP074089 and the data of *S. aureus* under Accession No. SRP074912. For *E. coli* between 405 and 1,260 Mb clean data (793 Mb on average) were obtained for each individual and for *S. aureus* between 800 and 1,556 Mb clean data (1,098 Mb on average) obtained.

We quality-filtered Illumina reads and generated a stringent SNP dataset by applying a strict minimum coverage filter. All unique regions were assessed to identify specific SNPs and indels. A total of 168,720 SNPs were identified from the 45 *E. coli* strains, and 83,642 SNPs were identified from the 45 *S. aureus* strains. SNP densities were approximately one per 23 bp in *E. coli* and one per 41 bp in *S. aureus*, which were expected to be sufficient for the identification of genomic regions associated with the traits of interest.

### Genetic Analysis of Microbial Growth

We employed a GWAS of the genomes of the 45 *E. coli* and 45 *S. aureus* strains to identify polymorphisms that were associated with different growth phenotypes (**Supplementary Table S5**). **Supplementary Figures S2–S4** showed Manhattan plots for the significant SNPs in *E. coli* that were identified in monoculture and co-culture, respectively. By plotting genomic locations against -log_10_ (*P*), 66 SNPs were found to be beyond the genome-wide critical thresholds determined after Bonferroni correction in *E. coli* at multiple time points of the 168,720 SNPs. Of those, 11 and 55 SNPs were associated with *E. coli* growth in monoculture and co-culture, respectively (**Supplementary Table S6**), which in total were mapped to 36 genes by annotation. Similarly, we drew Manhattan plots for the significant SNPs for growth in *S. aureus* (**Supplementary Figures S5–S7**). In monoculture, 54 SNPs were identified, whereas in co-culture, 57 SNPs identified, totaling to 111 significant SNPs mapped to 54 genes (**Supplementary Table S6**). In **Supplementary Table S7**, nine and eight indels were associated with *E. coli* growth in monoculture and co-culture, respectively, while such numbers of SNPs were nine and 11 for *S. aureus* growth.

**FIGURE 2:**
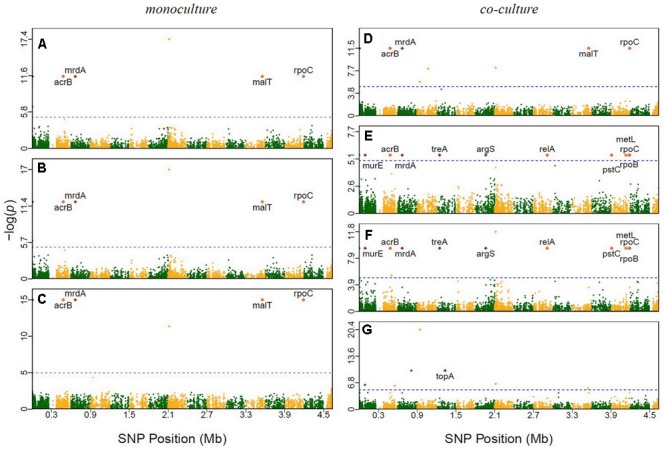
Manhattan plots of GWAS results for *E. coli* grown in monoculture and co-culture. The *x*-axis showed SNP positions (Mb) and the *y*-axis was the –log(*P*-value) resulting from the association test. Each dot in the plot represented an SNP, and a reference line was used on the *y*-axis to reflect genome-wide significance. Significant SNP identified in previous evolutionary studies were highlighted. **(A)** Time point 2; **(B)** time point 7; **(C)** time point 14; **(D)** time point 1; **(E)** time point 6; **(F)** time point 10; **(G)** time point 11.

**FIGURE 3:**
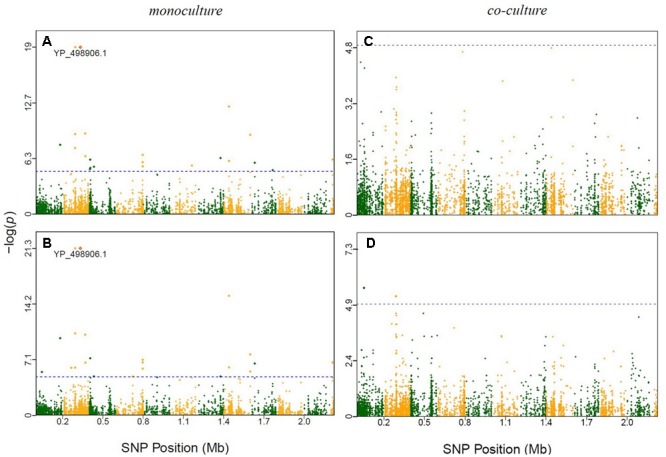
Manhattan plots of GWAS results for *S. aureus* in monoculture and co-culture. The *x*-axis showed SNP positions (Mb) and the *y*-axis was the –log(*P*-value) resulting from the association test. Each dot in the plot represented an SNP, and a reference line was used on the *y*-axis to reflect genome-wide significance. Significant SNP identified in previous evolutionary studies were highlighted. **(A)** Time point 4; **(B)** time point 5; **(C)** time point 4; **(D)** time point 5.

It is interesting to see that many of the significant SNPs detected from GWAS distributed in the regions of candidate genes involved in metabolism and regulation, and in hypothetical genes and intergenic regions (**Supplementary Table S6**). In *E. coli*, 66 SNPs detected in coding sequences could be divided into 20 non-synonymous and 16 synonymous SNPs. Twelve significant genes, such as murE, treA, argS, and relA, which had been identified in previous evolutionary studies (**Figure 2** and **Table 1**). In *S. aureus*, 35 out of 111 polymorphic changes result in amino acid alterations. One hypothetical gene (SAOUHSC_00316) was predicted to function as MepB protein which was reported to play a role in responding to antimicrobials in monoculture (**Figure 3**) (Agah et al., 2014); We didn’t find any genes which were reported to be correlated with evolution in co-culture.

**Table 1:** Mutations discovered in the sequenced genomes of *Escherichia coli* strains.

Gene ID	Gene (protein)	Position	Mutation	Affected codon	Function	Reference
**Co-culture**						
b0085	*murE*	94633	C<->T	CTG<->TTG	Peptidoglycan biosynthetic process	Herring et al., 2006
b0462	*acrB*	481615	C<->T	GGC<->GAC	peptidoglycan-based cell wall	Minty et al., 2011
		481849	G<->A	CCT<->CTT		
		482766	C<->T	CTG<->CTA		
b0635	*mrdA*	667835	C<->T	CGT<->CAT	Cell elongation	Conrad et al., 2009
		667511	A<->G	ATT<->ACT		
b1197	*treA*	1246976	T<->C	GAA<->GGA;	Trehalase	Conrad et al., 2009
b1274	*topA*	4636737	G<->A	GGC<->GGT	DNA topoisomerase type I	Barrick et al., 2009
b1876	*argS*	1961241	C<->T	CTG<->TTG;	Arginine tRNA synthetase	Conrad et al., 2009
b2784	*relA*	2913181	A<->T	ATT<->ATA	Stringent factor	Conrad et al., 2009
		2913520	T<->C	CAA<->CAG		
b3418	*malT*	3554135	T<->A	TGG<->AGG	Positive regulator of mal regulon	Barrick et al., 2009
		3554708	G<->A	GGG<->AGG		
b3727	*pstC*	3910077	G<->T	CTG<->ATG;	peptidoglycan-based cell wall	Minty et al., 2011
b3940	*metL*	4130938	G<->C	GTG<->GTC	Methionine biosynthetic process	LaCroix et al., 2015
		4130941	C<->T	GCC<->GCT		
		4131169	T<->C	ATT<->ATC		
		4131221	T<->C	TTG<->CTG		
		4131244	T<->C	GGT<->GGC		
		4131343	C<->T	AGC<->AGT		
		4131643	C<->T	GTC<->GTT		
		4131757	C<->T	TTC<->TTT		
b3987	*rpoB*	4181691	G<->A	CTG<->CTA	Transcription	Herring et al., 2006; Conrad et al., 2009; LaCroix et al., 2015
b3988	*rpoC*	4189406	G<->A	GTG<->ATG	Transcription	Herring et al., 2006; Conrad et al., 2009
		4185634	T<->A	ACT<->ACA		
		4185886	G<->A	AAG<->AAA		
		4186153	G<->A	CTG<->CTA		
		4186975	T<->C	GCT<->GCC		
		4189526	C<->T	CTG<->TTG		
**Monoculture**						
b0462	*acrB*	481615	C<->T	GGC<->GAC	peptidoglycan-based cell wall	Minty et al., 2011
b0635	*mrdA*	667835	C<->T	CGT<->CAT;	Cell elongation	Conrad et al., 2009
b3418	*malT*	3554135	T<->A	TGG<->AGG	Positive regulator of mal regulon	Barrick et al., 2009
		3554708	G<->A	GGG<->AGG		
b3988	*rpoC*	4189406	G<->A	GTG<->ATG	Transcription	Herring et al., 2006; Conrad et al., 2009

Indels were mostly distributed in some hypothetical genes (**Supplementary Table S8**). In *E. coli*, one indel was found to affect the growth performance both in co-culture and monoculture. This indel located between gene *eutB* (ID = b2441) and *intZ* (ID = b2442). Ethanolamine ammonia-lyase encoded by *eutB* was involved in the pathway ethanolamine degradation, which is part of amine and polyamine degradation. Integrase (encoded by *intZ*) was necessary for integration of the phage into the *E. coli* genome by site-specific recombination. In co-culture of *S. aureus* the indel located between two hypothetical genes (SAOUHSC_02436 and 02437). One was predicted to function as aerobactin biosynthesis protein and the other function as putative e for IS1272.

## Discussion

It has been recognized that, in an ecological community composed of multiple species, the existence of one species forms the environment of any other species that interact with it (Brown et al., 2001; Brockhurst et al., 2003). How a species responds to such a biotic environment in the community, ultimately leading to its evolution, has been a topic of intense interest to ecological evolutionary biologists (Turcotte et al., 2012; Andrade-Domínguez et al., 2014). In a study of community ecology, Lawrence et al. (2012) suggested that some species have evolved to consume waste products of other species, a phenomenon thought to be widespread in nature (Turcotte et al., 2012). In another study based on adaptive laboratory evolution, LaCroix et al. (2015) identified specific mutations that cause phenotypic optimization essential for the coexistence of microorganisms in a community.

Despite the role of ecological interactions in the adaptation of species, a detailed picture of its underlying genetic mechanisms is unknown (Johansson, 2008). By choosing two bacterial species, *E. coli* and *S. aureus*, we performed an experiment of ecological evolution to map genes that modulate ecological interactions in a microbial community. This experiment takes advantage of next-generation sequencing technologies that enable comprehensive analyses of microbial genomes (Forde and O’Toole, 2013). Moreover WGS has facilitated the discovery of multiple metabolic pathways that underlie the evolution of various phenotypes in *E. coli* (LaCroix et al., 2015). Using WGS, Ojeda et al. (2014) conducted a population genomics study of fungal symbionts using 304 samples from 36 populations. By performing genome resequencing of *E. coli*, (Minty et al., 2011) identified multiple evolutionary changes that increase the tolerance of this bacteria to isobutanol in an evolution experiment. All these previous studies have stimulated us to develop a GWAS to characterize the genetic basis of microbial growth in a competitive environment. GWAS has proven to be a powerful tool for uncovering new details of genetic control for complex traits, although its application to bacteria is still in infancy (Alam et al., 2014; Read and Massey, 2014).

In the experiment of ecological evolution with *E. coli* and *S. aureus*, we cultured 45 pairs of strains, drawn from each species, individually in isolation and jointly in terms of interspecific pairs. This experiment can address the fundamental question of whether and how the growth of a strain is affected by the co-existence of its partner. Through GWAS, we have identified a number of genetic loci for microbial growth in monoculture and co-culture. Many of these SNPs were detected to reside in the regions of candidate genes with known functions (**Figure 2**). For example, a SNP detected in co-culture was found to be at *relA*, a well-known regulatory gene that drives adaptive evolution of *E. coli* K-12 MG1655 in lactate minimal media (Conrad et al., 2009). Three of the previously reported genes, *murE* (Herring et al., 2006), *acrB* (Minty et al., 2011), and *pstC* (Minty et al., 2011), affect peptidoglycan metabolism during adaptation to glycerol-based growth media and isobutanol stress, respectively. The *topA* gene, which encodes a DNA topoisomerase, and *malT* gene, which functions in positive regulator of mal regulon, was identified in a long-term experiment of genome evolution and adaptation with *E. coli* (Barrick et al., 2009). Two genes – *rpoB* (Herring et al., 2006; Conrad et al., 2009; LaCroix et al., 2015) and *rpoC* (Herring et al., 2006; Conrad et al., 2009; LaCroix et al., 2015) – were shown to affect the regulation of RNA transcription in studies of laboratory evolution. Other four genes (*mrdA, treA, argS*, and *relA*) were also reported in a research about *E. coli* K-12 MG1655 undergoing short-term laboratory evolution in lactate minimal media (Conrad et al., 2009). The gene *metL*, which affects methionine biosynthesis (LaCroix et al., 2015), was involved in carbohydrate catabolism in a study of laboratory evolution. Some SNPs in specific genes were detected in this study to illustrate the genetic architecture of ecological interactions of *E. coli* and *S. aureus* in co-culture. Nevertheless, results reported from this genome-wide association study are hypothesis-generating and will require further functional validation.

Our integration of evolution experiment and GWAS with microbial populations provides a platform to address many important questions about the origin of natural interactions (Andrade-Domínguez et al., 2014). Moreover, the findings from this integration are useful for facilitating a detailed understanding of the dynamics of infectious diseases, the establishment and function of microbial communities, and the decline of microbial lineages. This understanding will promote the identification of key parameters and relationships that contribute to complex microbiological systems (Hibbing et al., 2010). The tree-hole species tend to use similar resources, which may lead to competition among microbes. Thus, a deep understanding of whether and how bacterial species compete or cooperate may provide new insight into their long-term co-adaptation and the degree of their niche overlapping (Ren et al., 2015). In clinical practice, knowledge about evolutionary changes of ecological interactions between different bacteria is necessary for designing effective antibacterial therapies (Turcotte et al., 2012).

## Data Accessibility

The raw sequence data of *E. coli* generated in this study were deposited in the NCBI short reads archive under Accession No. SRP074089 and the data of *S. aureus* under Accession No. SRP074912.

## Author Contributions

XH and RW conceived and designed the experiments. NC, JZ, and JW performed the experiments. YJ, MY, and LJ analyzed the data. XH and YJ contributed reagents/materials/analysis tools. XH and RW wrote the paper.

## Conflict of Interest Statement

The authors declare that the research was conducted in the absence of any commercial or financial relationships that could be construed as a potential conflict of interest.

## Footnotes

1 http://faculty.washington.edu/tathornt/software/QROADTRIPS/

## Supplementary Material

FIGURE S1 Design of the evolution experiments. (i) Isolates were propagated from frozen stocks that each contained a single starting genotype of a species. (ii) Experiments began with the growth of each species in monoculture and co-culture. (iii) To stimulate growth and promote adaptation to laboratory conditions, each culture was diluted 20-fold in fresh medium twice weekly for 8 weeks. Flasks were agitated to prevent biofilm formation and maintain spatial homogeneity. (iv) Single colonies from each species were isolated for the growth assays described in the main text.

FIGURE S2 Manhattan plots for the significant SNPs in *Escherichia coli* identified in monoculture. Genomic location was plotted against -log_10_ (*P*), with 86 SNPs identified at multiple time points. Each dot corresponds to a single SNP.

FIGURE S3 Manhattan plots for the significant SNPs in *E. coli* identified in co-culture. Genomic location was plotted against -log_10_ (*P*), with 1598 SNPs identified at multiple time points. Time point 1–4,6–8.

FIGURE S4 Manhattan plots for the significant SNPs in *E. coli* identified in co-culture. Genomic location was plotted against -log_10_ (*P*), with 1598 SNPs identified at multiple time points. Time point 9–11,13,14,16.

FIGURE S5 Manhattan plots for the significant SNPs in *Staphylococcus aureus* identified in monoculture. Genomic location is plotted against -log_10_ (*P*), with 407 SNPs identified at multiple time points. Each dot corresponds to a single SNP.

FIGURE S6 Manhattan plots for the significant SNPs in *S. aureus* identified in co-culture. Genomic location is plotted against -log_10_ (*P*), with 134 SNPs identified at multiple time points. Each dot corresponds to a single SNP. Time point 1–3,5–8.

FIGURE S7 Manhattan plots for the significant SNPs in *S. aureus* identified in co-culture. Genomic location is plotted against -log_10_ (*P*), with 134 SNPs identified at multiple time points. Each dot corresponds to a single SNP. Time point 9–16.

TABLE S1 Strain IDs.

TABLE S2 Resequencing statistics for *E. coli*.

TABLE S3 Resequencing statistics for *S. aureus*.

TABLE S4 Average sequencing depth and coverage for *E. coli* and *S. aureus*.

TABLE S5 Growth performance of each species in evolution experiments.

TABLE S6 Gene annotation for *E. coli* and *S. aureus* SNPs associated with growth performance.

TABLE S7 Indels associated with growth phenotypes.

TABLE S8 Gene annotation for *E. coli* and *S. aureus* indels associated with growth performance.
