# Supplementary figures and images for: Bacterial Genetic Architecture of Ecological Interactions in Co-culture by GWAS-Taking Escherichia coli and Staphylococcus aureus as an Example
Source: Front Microbiol. 2017 Nov 27;8:2332. doi: 10.3389/fmicb.2017.02332 (PMC5712204; doi:10.3389/fmicb.2017.02332)

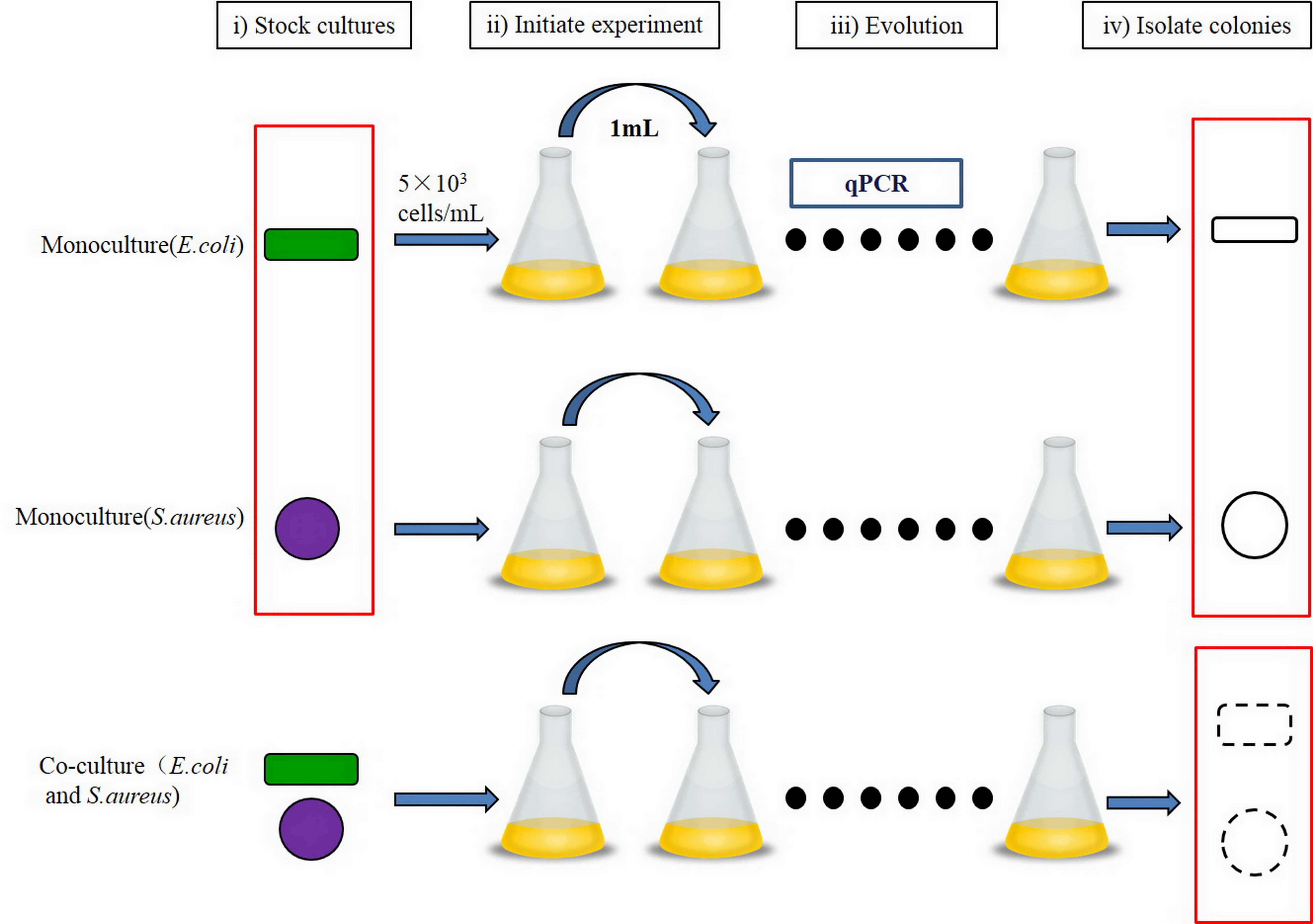

Supplement: FIGURE S1 — Design of the evolution experiments. (i) Isolates were propagated from frozen stocks that each contained a single starting genotype of a species. (ii) Experiments began with the growth of each species in monoculture and co-culture. (iii) To stimulate growth and promote adaptation to laboratory conditions, each culture was diluted 20-fold in fresh medium twice weekly for 8 weeks. Flasks were agitated to prevent biofilm formation and maintain spatial homogeneity. (iv) Single colonies from each species were isolated for the growth assays described in the main text. [file Image_1.JPEG]

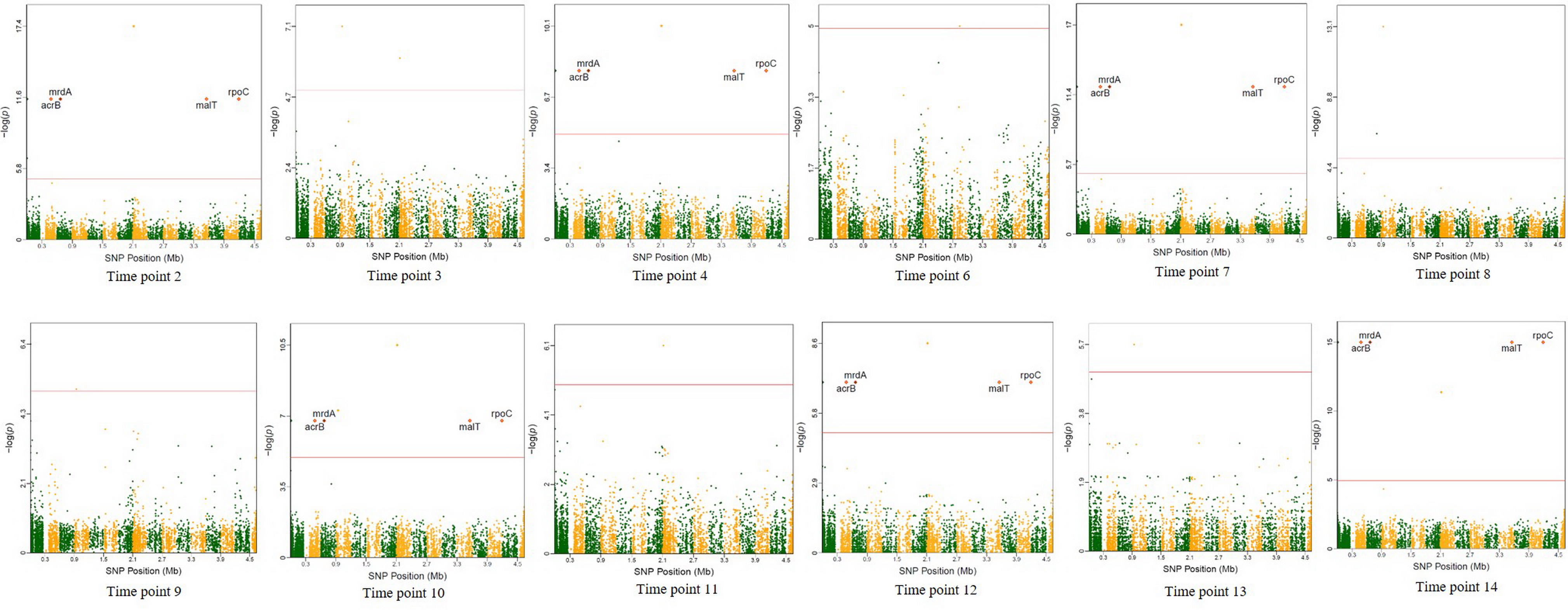

Supplement: FIGURE S2 — Manhattan plots for the significant SNPs in Escherichia coli identified in monoculture. Genomic location was plotted against -log10 (P), with 86 SNPs identified at multiple time points. Each dot corresponds to a single SNP. [file Image_2.JPEG]

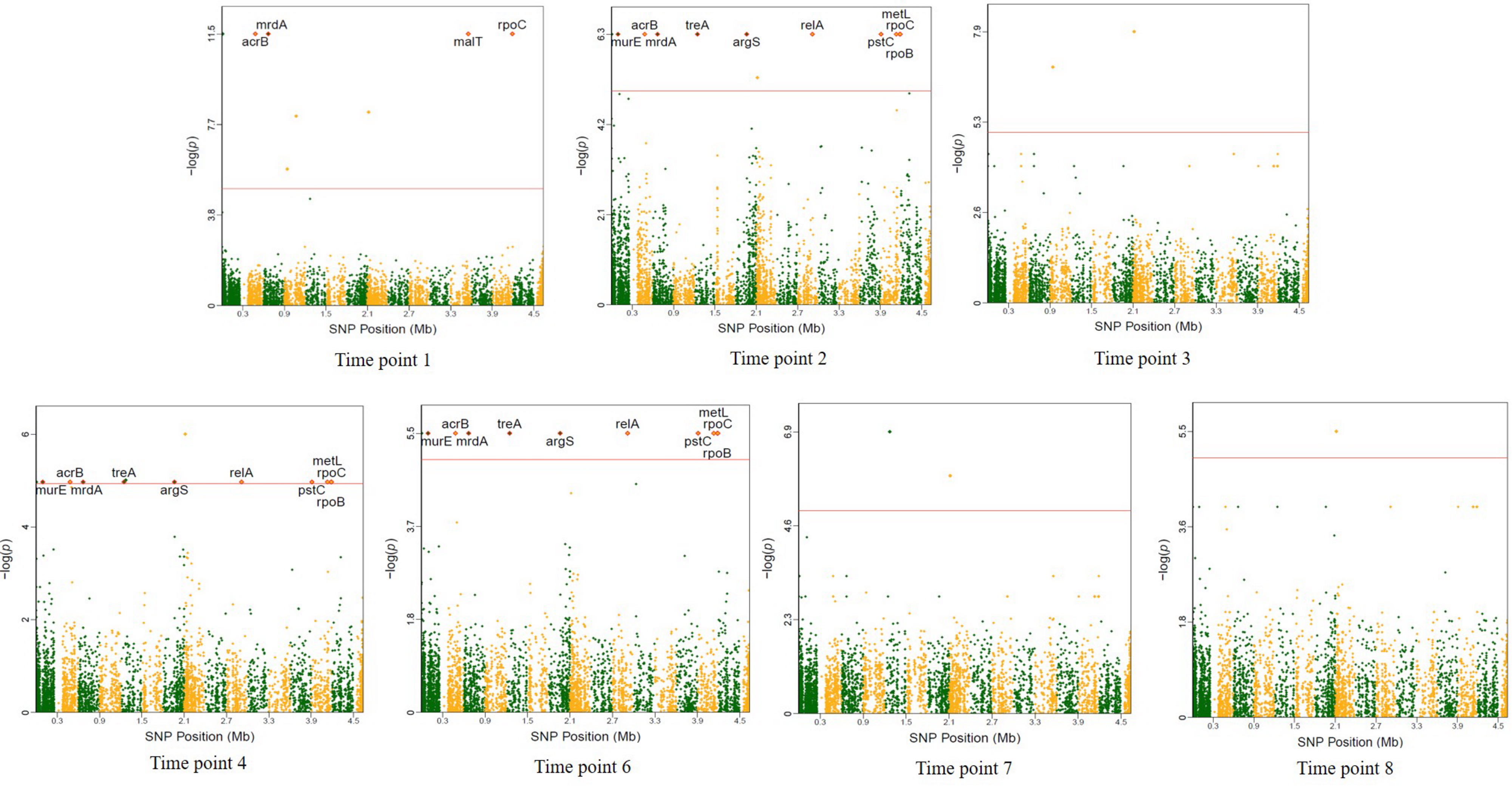

Supplement: FIGURE S3 — Manhattan plots for the significant SNPs in E. coli identified in co-culture. Genomic location was plotted against -log10 (P), with 1598 SNPs identified at multiple time points. Time point 1–4,6–8. [file Image_3.JPEG]

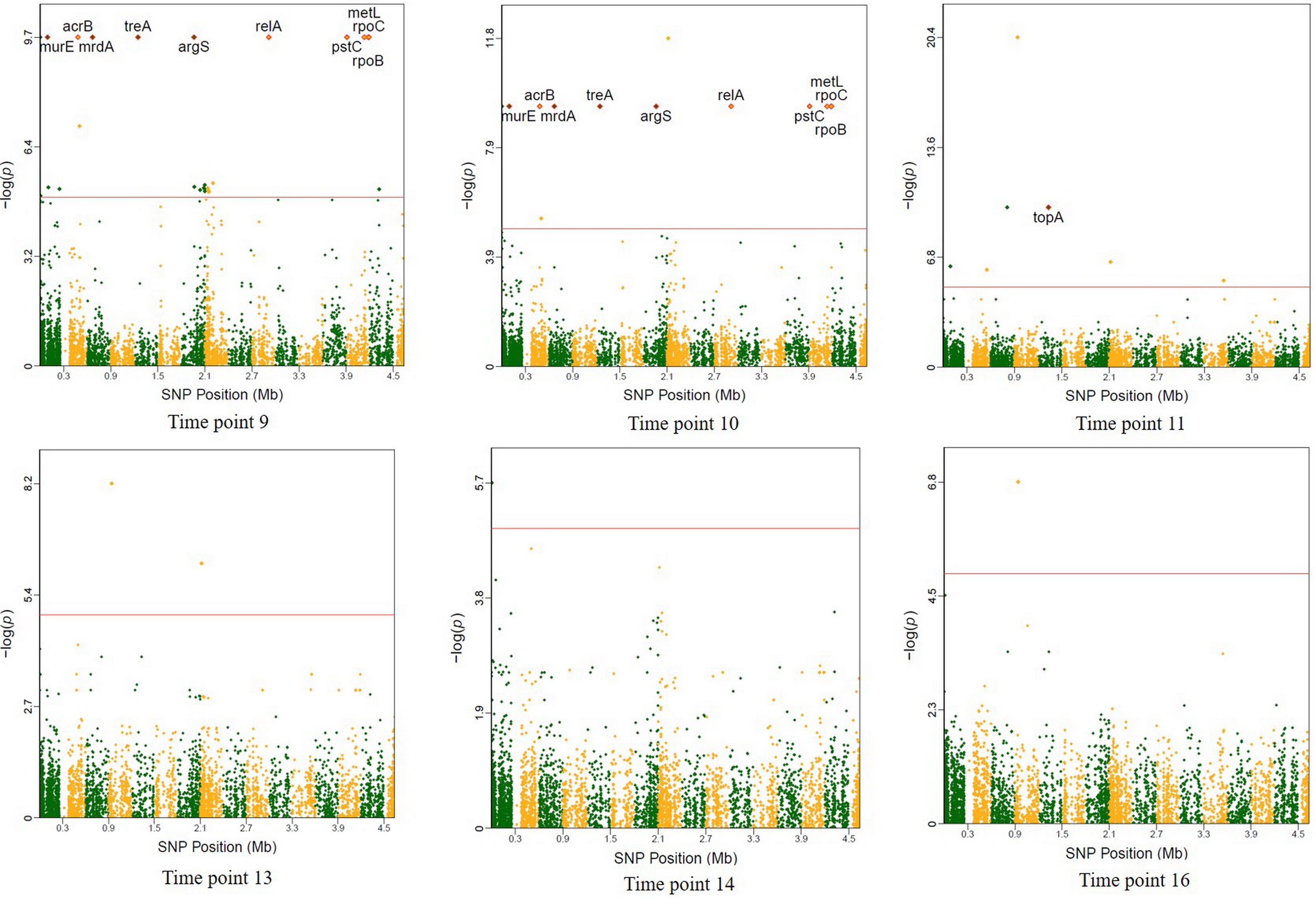

Supplement: FIGURE S4 — Manhattan plots for the significant SNPs in E. coli identified in co-culture. Genomic location was plotted against -log10 (P), with 1598 SNPs identified at multiple time points. Time point 9–11,13,14,16. [file Image_4.JPEG]

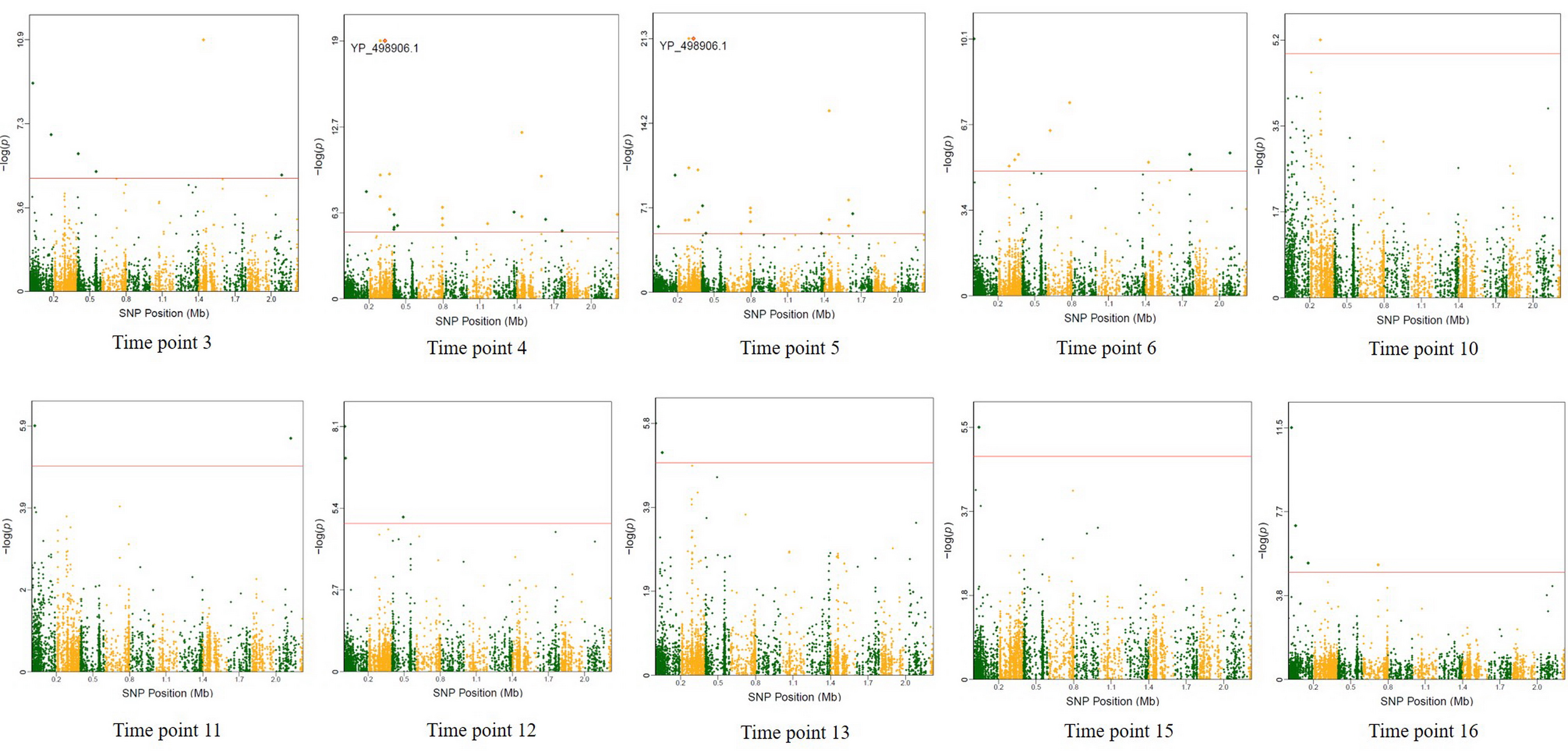

Supplement: FIGURE S5 — Manhattan plots for the significant SNPs in Staphylococcus aureus identified in monoculture. Genomic location is plotted against -log10 (P), with 407 SNPs identified at multiple time points. Each dot corresponds to a single SNP. [file Image_5.JPEG]

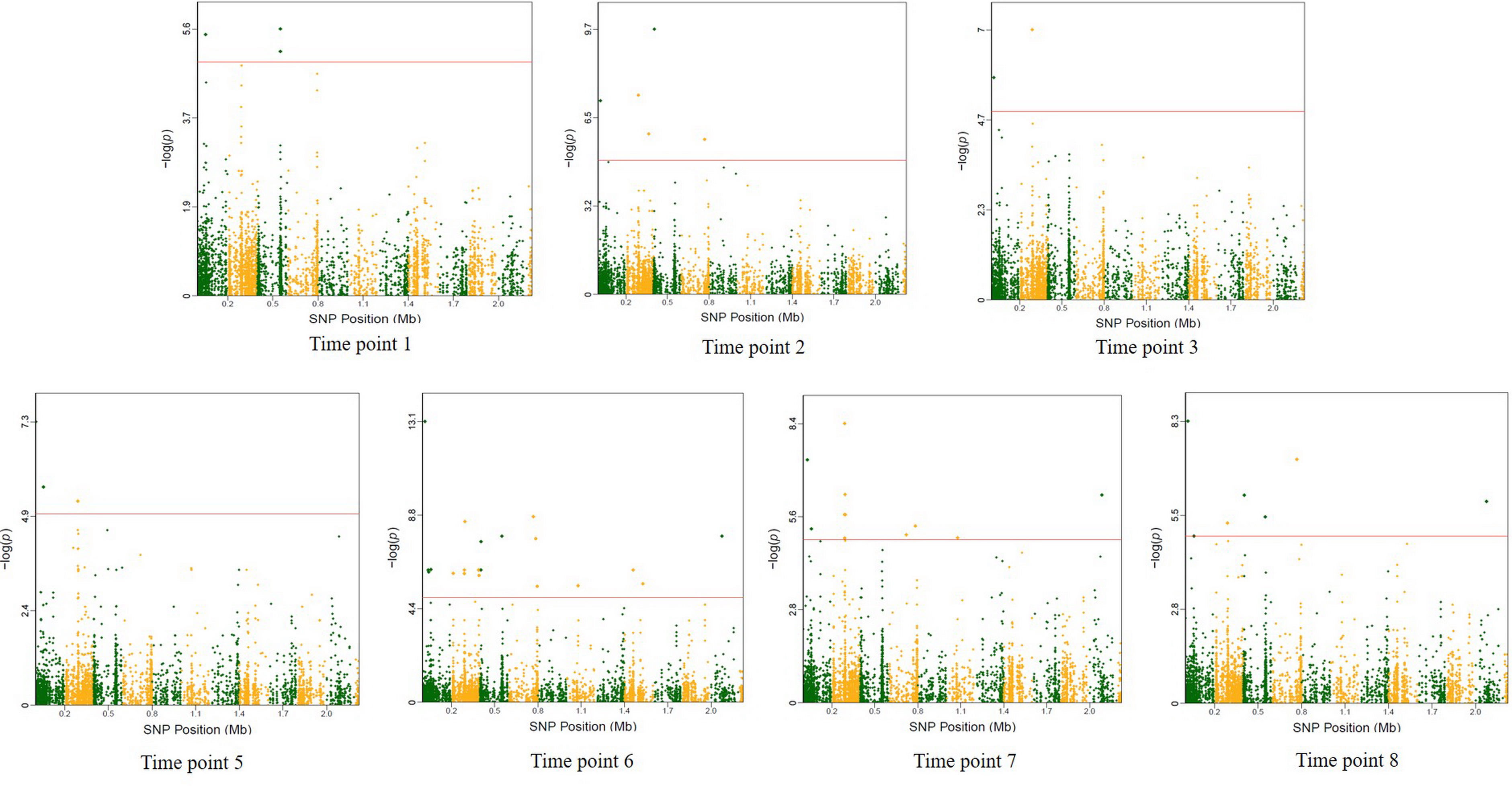

Supplement: FIGURE S6 — Manhattan plots for the significant SNPs in S. aureus identified in co-culture. Genomic location is plotted against -log10 (P), with 134 SNPs identified at multiple time points. Each dot corresponds to a single SNP. Time point 1–3,5–8. [file Image_6.JPEG]

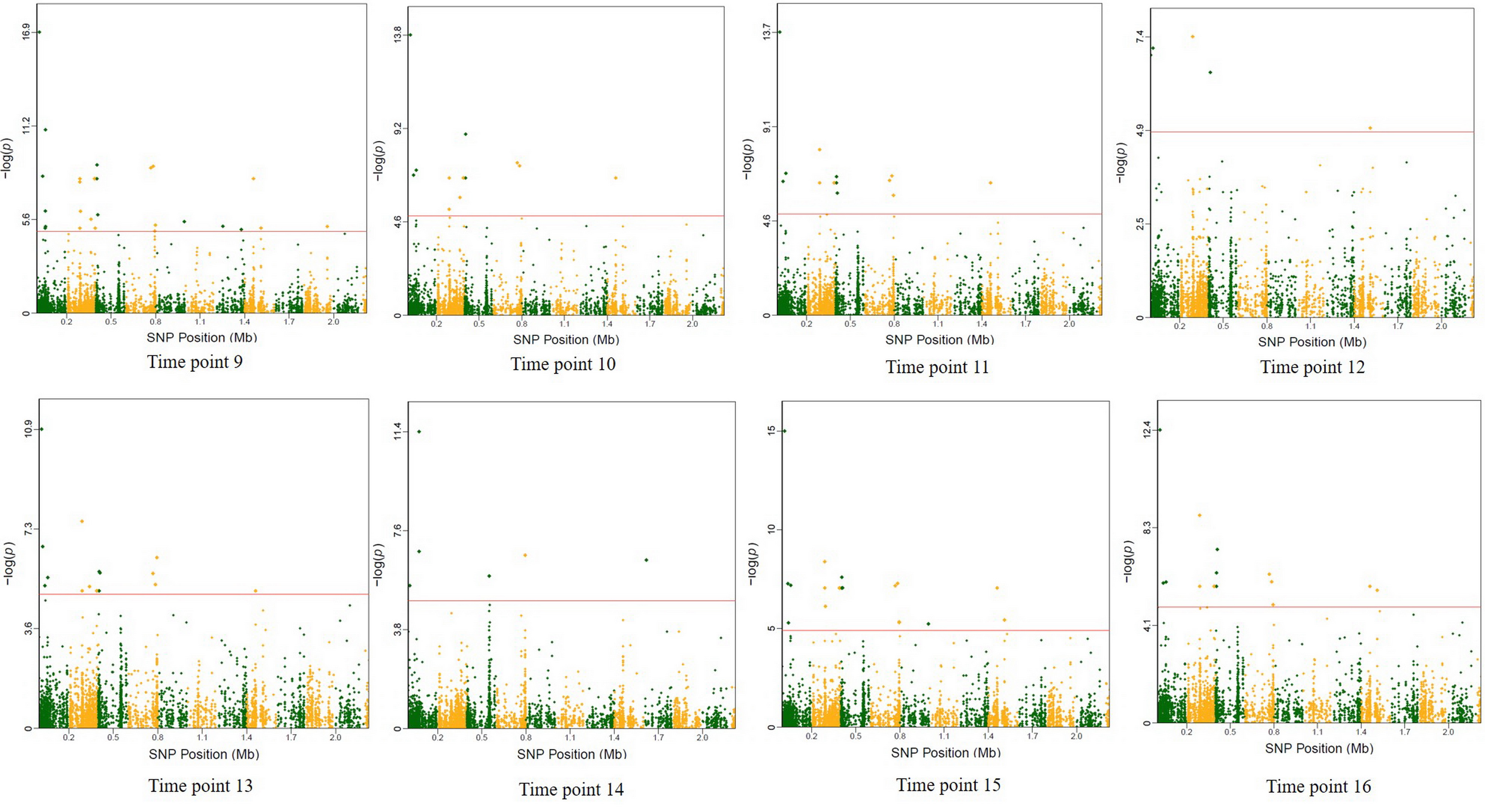

Supplement: FIGURE S7 — Manhattan plots for the significant SNPs in S. aureus identified in co-culture. Genomic location is plotted against -log10 (P), with 134 SNPs identified at multiple time points. Each dot corresponds to a single SNP. Time point 9–16. [file Image_7.JPEG]
