# Supplementary material for: Bacterial Genetic Architecture of Ecological Interactions in Co-culture by GWAS-Taking Escherichia coli and Staphylococcus aureus as an Example
Source: Front Microbiol. 2017 Nov 27;8:2332. doi: 10.3389/fmicb.2017.02332 (PMC5712204; doi:10.3389/fmicb.2017.02332)
Supplement: TABLE S2 — Resequencing statistics for E. coli. [file Table_2.DOCX]

**Table S2 Resequencing statistics for *E.coli***

| **Sample ID** | **Insert size (bp)** | **Reads length (bp)** | **Raw data (Mb)** | **Filtered reads (%)** | **Clean data (Mb)** | **Clean data Q20(%)** | **Clean data Q30(%)** |
| --- | --- | --- | --- | --- | --- | --- | --- |
| D1 | 500 | (100:100) | 992 | 30.18 | 693 | 91.75 | 77.57 |
| D2 | 500 | (100:100) | 1,421 | 27.95 | 1,024 | 95.07 | 86.00 |
| D3 | 500 | (100:100) | 1,015 | 31.37 | 696 | 91.18 | 78.30 |
| D4 | 500 | (100:100) | 1,332 | 30.15 | 932 | 90.34 | 78.48 |
| D5 | 500 | (100:100) | 1,710 | 28.20 | 1,228 | 95.17 | 86.92 |
| D6 | 500 | (100:100) | 682 | 27.17 | 497 | 94.28 | 82.75 |
| D7 | 500 | (100:100) | 720 | 33.01 | 483 | 91.42 | 76.84 |
| D8 | 500 | (100:100) | 930 | 25.82 | 690 | 92.02 | 79.76 |
| D9 | 500 | (100:100) | 1,060 | 24.68 | 798 | 92.36 | 80.12 |
| D10 | 500 | (100:100) | 853 | 30.27 | 595 | 91.82 | 77.81 |
| D11 | 500 | (125:125) | 935 | 18.47 | 762 | 94.80 | 88.80 |
| D12 | 500 | (100:100) | 1,344 | 26.64 | 986 | 95.21 | 87.08 |
| D13 | 500 | (100:100) | 1,586 | 26.74 | 1,162 | 95.27 | 87.15 |
| D14 | 500 | (100:100) | 945 | 22.37 | 733 | 92.95 | 81.26 |
| D15 | 500 | (100:100) | 815 | 19.87 | 653 | 95.37 | 85.25 |
| D16 | 500 | (100:100) | 854 | 28.04 | 615 | 91.50 | 78.60 |
| D17 | 500 | (100:100) | 1,070 | 28.60 | 764 | 95.04 | 85.96 |
| D18 | 500 | (100:100) | 1,316 | 26.83 | 963 | 95.17 | 86.36 |
| D19 | 500 | (100:100) | 895 | 28.24 | 643 | 91.44 | 78.50 |
| D20 | 500 | (100:100) | 831 | 23.76 | 633 | 93.47 | 81.72 |
| D21 | 500 | (100:100) | 1,418 | 27.25 | 1,032 | 95.28 | 87.16 |
| D22 | 500 | (100:100) | 1,183 | 26.51 | 869 | 95.12 | 86.12 |
| D23 | 450 | (125:125) | 1,086 | 12.41 | 952 | 94.63 | 88.78 |
| D24 | 500 | (100:100) | 839 | 24.21 | 636 | 94.63 | 83.56 |
| D25 | 500 | (125:125) | 680 | 20.09 | 544 | 94.82 | 88.88 |
| D26 | 500 | (100:100) | 1,084 | 30.29 | 756 | 95.36 | 86.96 |
| D27 | 500 | (100:100) | 1,472 | 26.93 | 1,076 | 95.15 | 86.85 |
| D28 | 500 | (100:100) | 1,116 | 18.07 | 915 | 95.87 | 86.03 |
| D29 | 500 | (100:100) | 1,120 | 28.80 | 798 | 95.58 | 87.39 |
| D30 | 500 | (100:100) | 1,016 | 17.90 | 834 | 97.28 | 90.04 |
| D31 | 500 | (100:100) | 940 | 30.85 | 650 | 95.69 | 88.71 |
| D32 | 500 | (100:100) | 964 | 32.68 | 649 | 94.83 | 86.15 |
| D33 | 500 | (100:100) | 1,323 | 23.53 | 1,012 | 92.57 | 80.49 |
| D34 | 500 | (100:100) | 882 | 23.07 | 679 | 92.66 | 80.95 |
| D35 | 500 | (100:100) | 954 | 36.55 | 605 | 94.28 | 85.02 |
| D36 | 500 | (100:100) | 543 | 25.42 | 405 | 94.41 | 83.03 |
| D37 | 500 | (100:100) | 981 | 32.94 | 658 | 91.38 | 76.78 |
| D38 | 500 | (100:100) | 1,706 | 26.17 | 1,260 | 95.31 | 87.25 |
| D39 | 500 | (100:100) | 1,714 | 27.45 | 1,243 | 95.20 | 86.97 |
| D40 | 500 | (125:125) | 1,123 | 22.03 | 876 | 94.82 | 88.70 |
| D41 | 500 | (100:100) | 995 | 31.08 | 686 | 91.07 | 78.07 |
| D42 | 500 | (125:125) | 900 | 18.34 | 735 | 94.90 | 88.81 |
| D43 | 500 | (100:100) | 1,151 | 28.82 | 820 | 95.97 | 89.31 |
| D44 | 500 | (125:125) | 814 | 14.72 | 694 | 96.01 | 91.24 |
| D45 | 500 | (100:100) | 1,096 | 31.37 | 752 | 94.94 | 86.43 |
