# Supplementary material for: Bacterial Genetic Architecture of Ecological Interactions in Co-culture by GWAS-Taking Escherichia coli and Staphylococcus aureus as an Example
Source: Front Microbiol. 2017 Nov 27;8:2332. doi: 10.3389/fmicb.2017.02332 (PMC5712204; doi:10.3389/fmicb.2017.02332)
Supplement: TABLE S3 — Resequencing statistics for S. aureus. [file Table_3.DOCX]

**Table S2 Resequencing statistics of *S.aureus***

| **Sample ID** | **Insert size (bp)** | **Reads length (bp)** | **Raw data (Mb)** | **Filtered reads (%)** | **Clean data (Mb)** | **Clean data Q20(%)** | **Clean data Q30(%)** |
| --- | --- | --- | --- | --- | --- | --- | --- |
| J1 | 500 | (100:100) | 1,012 | 18.86 | 821 | 97.01 | 90.85 |
| J2 | 500 | (100:100) | 1,215 | 22.99 | 935 | 93.32 | 82.34 |
| J3 | 500 | (100:100) | 1,012 | 9.88 | 912 | 98.54 | 94.28 |
| J4 | 500 | (100:100) | 1,247 | 9.54 | 1,128 | 97.61 | 91.41 |
| J5 | 500 | (100:100) | 1,220 | 10.68 | 1,090 | 96.51 | 88.97 |
| J6 | 500 | (100:100) | 1,166 | 8.78 | 1,064 | 97.46 | 90.96 |
| J7 | 500 | (100:100) | 1,094 | 8.99 | 996 | 97.37 | 90.67 |
| J8 | 500 | (100:100) | 1,272 | 9.31 | 1,153 | 97.74 | 92.00 |
| J9 | 500 | (100:100) | 1,099 | 15.29 | 931 | 97.53 | 92.29 |
| J10 | 500 | (100:100) | 1,159 | 13.04 | 1,008 | 97.67 | 92.30 |
| J11 | 500 | (100:100) | 1,468 | 14.44 | 1,256 | 97.48 | 91.83 |
| J12 | 500 | (100:100) | 1,235 | 8.89 | 1,125 | 96.58 | 88.98 |
| J13 | 500 | (100:100) | 963 | 9.62 | 871 | 96.3 | 88.34 |
| J14 | 500 | (100:100) | 1,237 | 9.75 | 1,116 | 96.28 | 88.32 |
| J15 | 500 | (100:100) | 1,026 | 10.02 | 923 | 97.38 | 90.73 |
| J16 | 500 | (100:100) | 1,009 | 9.06 | 917 | 98.49 | 94.04 |
| J17 | 500 | (100:100) | 1,134 | 10.24 | 1,018 | 96.63 | 89.24 |
| J18 | 500 | (100:100) | 1,173 | 9.30 | 1,064 | 97.76 | 92.11 |
| J19 | 500 | (100:100) | 1,088 | 11.45 | 964 | 97.01 | 89.67 |
| J20 | 500 | (100:100) | 1,220 | 9.94 | 1,099 | 96.56 | 89.03 |
| J21 | 500 | (100:100) | 1,015 | 16.51 | 848 | 96.07 | 87.04 |
| J22 | 500 | (100:100) | 1,194 | 13.93 | 1,028 | 98.18 | 94.17 |
| J23 | 500 | (100:100) | 1,056 | 18.80 | 858 | 97.52 | 92.65 |
| J24 | 500 | (125:125) | 1,378 | 4.73 | 1,312 | 96.18 | 92.09 |
| J25 | 500 | (125:125) | 1,668 | 6.69 | 1,556 | 96.3 | 92.37 |
| J26 | 500 | (100:100) | 1,120 | 28.52 | 800 | 95.58 | 87.75 |
| J27 | 500 | (125:125) | 1,263 | 8.71 | 1,153 | 95.9 | 91.63 |
| J28 | 500 | (125:125) | 1,536 | 5.95 | 1,444 | 96.39 | 92.5 |
| J29 | 500 | (100:100) | 1,212 | 9.15 | 1,101 | 96.39 | 88.50 |
| J30 | 500 | (100:100) | 1,215 | 14.90 | 1,034 | 94.95 | 85.49 |
| J31 | 500 | (100:100) | 1,821 | 15.68 | 1,535 | 97.37 | 91.55 |
| J32 | 500 | (125:125) | 1,417 | 5.86 | 1,334 | 96.8 | 93.42 |
| J33 | 500 | (100:100) | 1,084 | 9.05 | 986 | 96.46 | 88.72 |
| J34 | 500 | (100:100) | 1,043 | 9.18 | 947 | 97.66 | 91.64 |
| J35 | 500 | (100:100) | 1,525 | 10.52 | 1,364 | 97.24 | 90.42 |
| J36 | 350 | (150:150) | 1,531 | 23.34 | 1,173 | 98.76 | 96.60 |
| J37 | 350 | (150:150) | 1,452 | 9.09 | 1,320 | 97.46 | 93.52 |
| J38 | 350 | (150:150) | 1,676 | 10.14 | 1,506 | 97.45 | 93.50 |
| J39 | 350 | (150:150) | 1,526 | 9.44 | 1,382 | 97.31 | 93.20 |
| J40 | 500 | (125:125) | 1,105 | 7.63 | 1,021 | 96.32 | 92.49 |
| J41 | 500 | (125:125) | 1,116 | 4.74 | 1,063 | 96.39 | 92.5 |
| J42 | 500 | (125:125) | 1,191 | 5.19 | 1,129 | 96.42 | 92.58 |
| J43 | 500 | (125:125) | 943 | 4.25 | 903 | 96.35 | 92.41 |
| J44 | 500 | (125:125) | 1,309 | 7.53 | 1,211 | 96.37 | 92.56 |
| J45 | 500 | (125:125) | 1,075 | 4.41 | 1,027 | 96.31 | 92.33 |
