# Supplementary material for: Bacterial Genetic Architecture of Ecological Interactions in Co-culture by GWAS-Taking Escherichia coli and Staphylococcus aureus as an Example
Source: Front Microbiol. 2017 Nov 27;8:2332. doi: 10.3389/fmicb.2017.02332 (PMC5712204; doi:10.3389/fmicb.2017.02332)
Supplement: TABLE S7 — Indels associated with growth phenotypes. [file Table_7.DOCX]

**Table S6 Indels associated with growth phenotypes**

| **Time points** | ***E.coli*** | |  | ***S.aureus*** | |
| --- | --- | --- | --- | --- | --- |
|  | **Monoculture** | **Co-culture** |  | **Monoculture** | **Co-culture** |
| 1 | 0 | 1  24  41  0  0  0  0  0  0  0  0  0  0  0  1  2 |  | 0 | 0 |
| 2 | 1 | 0 |  | 0 | 0 |
| 3 | 0 | 1 |  | 1 | 0 |
| 4 | 1 | 0 |  | 3 | 1 |
| 5 | 0 | 0 |  | 2 | 0 |
| 6 | 0 | 0 |  | 1 | 2 |
| 7 | 3 | 0 |  | 0 | 0 |
| 8 | 1 | 0 |  | 0 | 0 |
| 9 | 0 | 2 |  | 0 | 2 |
| 10 | 1 | 3 |  | 0 | 2 |
| 11 | 0 | 1 |  | 0 | 2 |
| 12 | 1 | 0 |  | 1 | 0 |
| 13 | 0 | 0 |  | 0 | 0 |
| 14 | 1 | 0 |  | 0 | 0 |
| 15 | 0 | 0 |  | 0 | 2 |
| 16 | 0 | 0 |  | 1 | 0 |
